# Single-cell profiling of dysfunctional NK cells in COVID-19: from immunization to acute infection and beyond

**DOI:** 10.3389/fimmu.2026.1972608

**Published:** 2026-10-20

**Authors:** Alessandro Frigo, Sara Terzoli, Paolo Marzano, Nicolò Coianiz, Michela Calvi, Lara Orlandi, Simone Balin, Valentina Cazzetta, Anna Carletti, Silvia Della Bella, Joanna Mikulak, Maciej Tarkowski, Matteo Bonfanti, Paolo Ferrari, Eugenia Ricciardelli, Clelia Peano, Agostino Riva, Giuseppe Testa, Antonio Voza, Francesca Calcaterra, Clara Di Vito, Domenico Mavilio

**Affiliations:** 1 Unit of Clinical and Experimental Immunology, IRCCS Humanitas Research Hospital, Rozzano, Milan, Italy; 2 Department of Medical Biotechnology and Translational Medicine, University of Milan, Milan, Italy; 3 Department of Biomedical Sciences, Humanitas University, Pieve Emanuele, Milan, Italy; 4 Luigi Sacco Department of Biomedical and Clinical Sciences DIBIC, University of Milan, Milan, Italy; 5 Human Technopole, Milan, Italy; 6 Institute of Genetics and Biomedical Research, UoS of Milan, National Research Council, Milan, Italy; 7 Department of Infectious Diseases, Luigi Sacco University Hospital, ASST Fatebenefratelli Sacco, Milan, Italy; 8 Department of Oncology and Hemato-Oncology, University of Milan, Milan, Italy; 9 Department of Biomedical Unit, IRCCS Humanitas Research Hospital, Milan, Italy

**Keywords:** COVID-19 severity, natural killer cells, NK-cell dysfunction, SARS-CoV-2, single-cell transcriptomics, vaccination

## Abstract

**Introduction:**

Vaccination and natural infection with Severe Acute Respiratory Syndrome CoronaVirus 2 (SARS-CoV-2) confer immune protection through distinct mechanisms, however, their effects on Natural Killer (NK) cell responses remain poorly understood, particularly in relation to COVID-19 disease severity. NK cells, key mediators of anti-viral immunity, have been implicated in coronavirus disease 2019 (COVID-19) pathogenesis, where severe disease is associated with hyperactivated yet dysfunctional NK-cell states that likely impair viral control and contribute to tissue damage.

**Methods:**

To define how vaccination and infection shape NK-cell biology, we performed longitudinal single-cell RNA sequencing (scRNA-seq) of NK cells from SARS-CoV-2-naïve individuals receiving two doses of the BNT162b2mRNA vaccine. These longitudinal vaccine profiles were integrated with cross-sectional scRNA-seq datasets from unvaccinated patients with acute COVID-19 and from independent convalescent individuals, all enrolled before vaccine introduction.

**Results:**

We identified distinct NK cell transcriptional programs associated with vaccination, acute infection and convalescence, with the most pronounced infection-related alterations observed in the severe form of the disease. These diverging transcriptional signatures correlate with differences in NK-cell subset composition and activation markers between vaccinated and infected individuals.

**Conclusions:**

Taken together, these findings provide a rationale for future studies examining whether vaccine-associated NK-cell transcriptional programs contribute to durable immune protection.

## 1 Introduction

Human Natural Killer (NK) cells are innate lymphocytes endowed with immune regulatory and cytotoxic features. In healthy individuals, NK cells account for 10-15% of circulating lymphocytes and are further divided into several subsets according to the expression of CD56 and CD16. The two main subsets, named CD56bright/CD16neg (CD56bright) and CD56dim/CD16pos (CD56dim) diverge in terms of functions and receptor repertoire, with CD56bright mostly involved in regulatory processes and CD56dim proficiently cytotoxic (1, 2). NK cells are crucial in controlling and resolving viral infections: independently from a prior antigen sensitization, they sense and clear viral-infected targets via direct cytotoxicity and antibody-dependent cell-mediated cytotoxicity (ADCC) (3, 4). Moreover, they orchestrate anti-viral responses by secreting interferons (IFNs) (4, 5).

Viral infected cells commonly downregulate MHC-I expression to escape T-cell mediated recognition. However, according to the “missing-self” hypothesis, NK cells can sense this downregulation and ultimately lyse target cells (6–8). This is finely regulated by inhibitory natural cytotoxicity receptors (NCRs), including NKG2A and killer-cell immunoglobulin-like receptors (KIRs) (9). Nonetheless, growing evidence suggests that activating NCRs are essential as well to elicit NK cell full potential: NKG2D, for example, specifically binds stress-induced molecules presented by MHC-I, while co-activating receptors concur in amplifying the response intensity. NK cells can sense several viral families, including herpesviruses, retroviruses and members of the coronaviruses. In turn, viruses evolved several strategies to escape immune surveillance and SARS-CoV-2, the causative agent of COVID-19, is a perfect case in point (10).

In late 2019, this novel beta coronavirus caused a pandemic respiratory infection. Its rapid spread, high pathogenicity, and ability to trigger asymptomatic infections underscored the need for immunological studies and vaccine development. COVID-19 patients exhibit NK cell depletion due to their pulmonary recruitment. In this scenario, it has been shown that NK cells play a dual role in viral defense and tissue damage (11, 12). Phenotypic and functional alterations induced by SARS-CoV-2 result in hyperactivated yet exhausted NK cells (13, 14). Indeed, while NK cells in acutely infected individuals exhibited markers of activation and inflammation (HLA-DR, CD69 and CX3CR1), SARS-CoV-2 can shape NK cells by increasing the expression of the inhibitory receptor NKG2A and reducing the ligands for the activating NKG2D (15–17). Moreover, NK cells from severe COVID-19 patients show compromised IFN-γ and TNF-α production and reduced cytotoxicity, with the final consequence of ineffective viral control.

This general impairment in NK cells during the acute SARS-CoV-2 infection is also accompanied by the expansion of CD56dim-low/CD16low-neg NK cells (18–20) and of hypofunctional CD56neg/CD16pos NK cells, typically present during chronic viral infection (21). Moreover, several studies reported the presence of adaptive-like NK cells, first described following human cytomegalovirus infection, directly correlating with a worsening prognosis in SARS-CoV-2 infected individuals (22–24).

The rapid SARS-CoV-2 spread, together with its variability in the clinical manifestations, ranging from asymptomatic to severe life-threatening forms, the increased susceptibility to severe disease in individuals with comorbidities and immunosuppression and the rapid emergence of novel variants urged the need for the prompt development of prophylactic vaccines. These vaccines, intended to protect single individuals, conferring herd-immunity and preventing the spreading of new variants of concern capable of breaking the immune surveillance, are composed by mRNA encoding for SARS-CoV-2 spike protein, a structural viral peptide aimed at safely triggering the humoral and the cellular immune responses, encapsulated in lipid nanoparticles (25–27).

However, whether mRNA vaccination induces transcriptional reprogramming in NK cells, and how these profiles differ from those elicited by natural infections, remain unknown. Prior studies have documented NK-cell activation markers post-vaccination (13, 28), but longitudinal single-cell transcriptomic characterization across vaccine doses and comparison to infection-induced states are lacking. Nonetheless, similarly to the natural anti-SARS-CoV-2 immunization, the effectiveness of such vaccines is reported to diminish over time, hence multiple administration regimens have been defined to sustain protective immunity. In this peculiar setting, the adaptive immune responses have been widely investigated. The third booster administration has been correlated with a more stable frequency of SARS-CoV-2 specific B and T cells, which may explain its longer-lasting effectiveness in preventing severe diseases (29). In addition to this, players of the innate immunity could be triggered by vaccine administration. In this context, several lines of evidence suggest that NK cell response, besides being relevant during the natural SARS-CoV-2 infection, could likely be stimulated and boosted by subsequential vaccine administrations (28, 30, 31).

Given these premises, we performed a longitudinal single-cell RNA sequencing (scRNA-seq) analysis to define the transcriptional programs induced in NK cells by subsequential vaccination versus natural infection, and to identify NK-cell states associated with disease severity. Specifically, we profiled the transcriptomic landscape of NK cells in SARS-CoV-2 naïve subjects following administration of two doses of the Pfizer-BioNTech BNT162b2 vaccine. Furthermore, we compared vaccine-induced NK cell profiles with those of SARS-CoV-2 infected individuals across different disease severity, both during the acute phase or after spontaneous recovery.

## 2 Results

### 2.1 Anti-SARS-CoV-2 mRNA-based vaccination does not affect NK cell distribution and dynamics

To investigate the impact of Pfizer-BioNTech BNT162b2 mRNA vaccine on circulating NK cells, peripheral blood samples were longitudinally collected from the “Vaccinated (V)” group, which includes 6 SARS-CoV-2-naïve healthy subjects who received two doses of vaccine. Samples were obtained at 5 different time points: the day before the vaccination (P0), and at +3 (P1) and +17 (P2) days following the first dose, and at +3 (P3) and +90 (P4) days after the second dose (**Figure 1A**) (32, 33). Peripheral blood mononuclear cells (PBMCs) were then subjected to 5’ scRNA-seq analysis.

**Figure 1:**
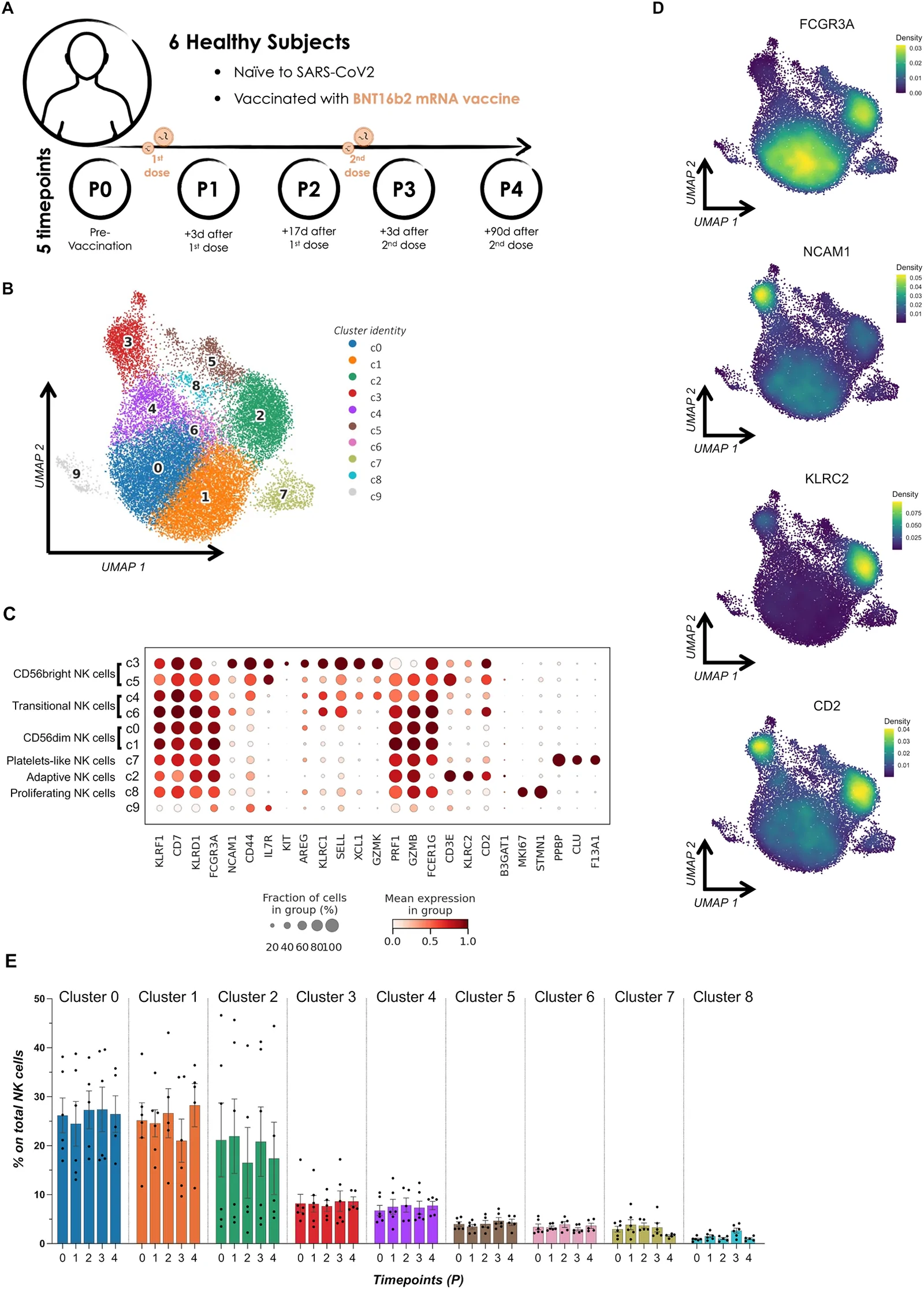
NK cell characterization in SARS-CoV-2-vaccinated subjects. **(A)** Schematic overview of the sampling timeline for the vaccinated (V) cohort. **(B)** *UMAP* visualization of 22’841 integrated PB NK cells from the 6 vaccinated subjects across the 5 time points. Cells are colored according to cluster (c) identity. C9 was excluded. **(C)** Dot plot showing the expression of genes used to annotate NK cell subsets within the different clusters. Dot dimension represents the percentage of expression while the color intensity indicates the mean gene expression. **(D)** Kernel Density plots showing the expression of the canonical markers used for NK cell annotation within the UMAP. **(E)** Bar plots showing the frequency (%) of NK cells within the different time points (P0-P4) across all NK cell clusters. Data are depicted as mean ± SEM, Kruskal-Wallis test for each cluster vs P0, n.s.

Unsupervised clustering analysis of 242’765 high-quality cells resulted in 37 clusters, manually annotated based on the expression of canonical genes (**Supplementary Figure 1A**). Within total PBMCs, NK cells were identified in clusters (c)2-22–34 based on the expression of canonical markers CD56 (*NCAM1)*, NKp80 (*KLRF1*) and CD16 (*FCGR3A)* along with the absence of other lineage markers (**Supplementary Figure 1B**) (34).

To further investigate the major NK cell subpopulations, a reclustering of c2-22–34 was performed, resulting in 10 clusters (**Figure 1B**). C9, expressing low levels of *KLRF1*, *CD7*, and *KLRD1*, was excluded from the downstream analysis. The remaining clusters, composed by a total of 22’841 cells, were annotated based on canonical NK cell markers. C3 and c5 were annotated as clusters of CD56bright NK cells, coherently with the high expression of *NCAM1*, *CD44, IL7R, KIT, KLRC1, SELL, XCL1 and GZMK*, with c5 weakly showing adaptive traits. CD56dim NK cells were identified in c0-1, both positive for the expression of *FCGR3A*, *PRF1, GZMB.* C4 and c6, expressing lower levels of CD56bright NK cell markers, but already displaying some genes typical of CD56dim NK cells, were annotated as transitional NK cells. C2 expressing *CD3E, KLRC2, CD2* were identified as adaptive NK cell, and c8 as *MKI67*^+^*STMN1*^+^ proliferating NK cells. C7, expressing high levels of *PPBP*, *CLU* and *F13A1*, was annotated as a cluster of NK-platelets aggregate (35), and excluded from further analyses (**Figure 1C**). Cluster annotation was confirmed by plotting the expression for major NK cell markers on the UMAP (**Figure 1D**).

Notably, the stratification of the V group by distinct time points before and after vaccination revealed no significant fluctuations in the frequencies of NK cell subsets. This observation suggests that mRNA-based vaccination does not promote the expansion of specific NK cell subsets (**Figure 1E**).

### 2.2 Dynamics of NK cell activation post-vaccine

To evaluate the effect of vaccine administration on NK cells, we analyzed the transcriptional changes across the major clusters (c1-4), which together encompassed the majority of NK cells in our dataset. Notably, all NK cell clusters showed increased responsiveness, measured as the number of differentially expressed genes (DEGs), particularly following the second vaccine dose. Clusters sharing similar biological features also displayed similar DEG dynamics.

Specifically, CD56dim NK cell clusters (c0, c1) exhibited a substantial number of DEGs both after the first vaccination (P1) and, more prominently, following the booster (P3). Contrariwise, CD56bright (c3) and transitional NK cells (c4) underwent the most pronounced transcriptional changes at P3, consistent with a stronger transcriptional impact on these populations. Last, adaptive CD56dim NK cells (c2) displayed a proper pattern, with higher DEGs at time points distant from both the first (P2) and the second doses (P4), although the magnitude of these changes was limited (**Figure 2A**).

**Figure 2:**
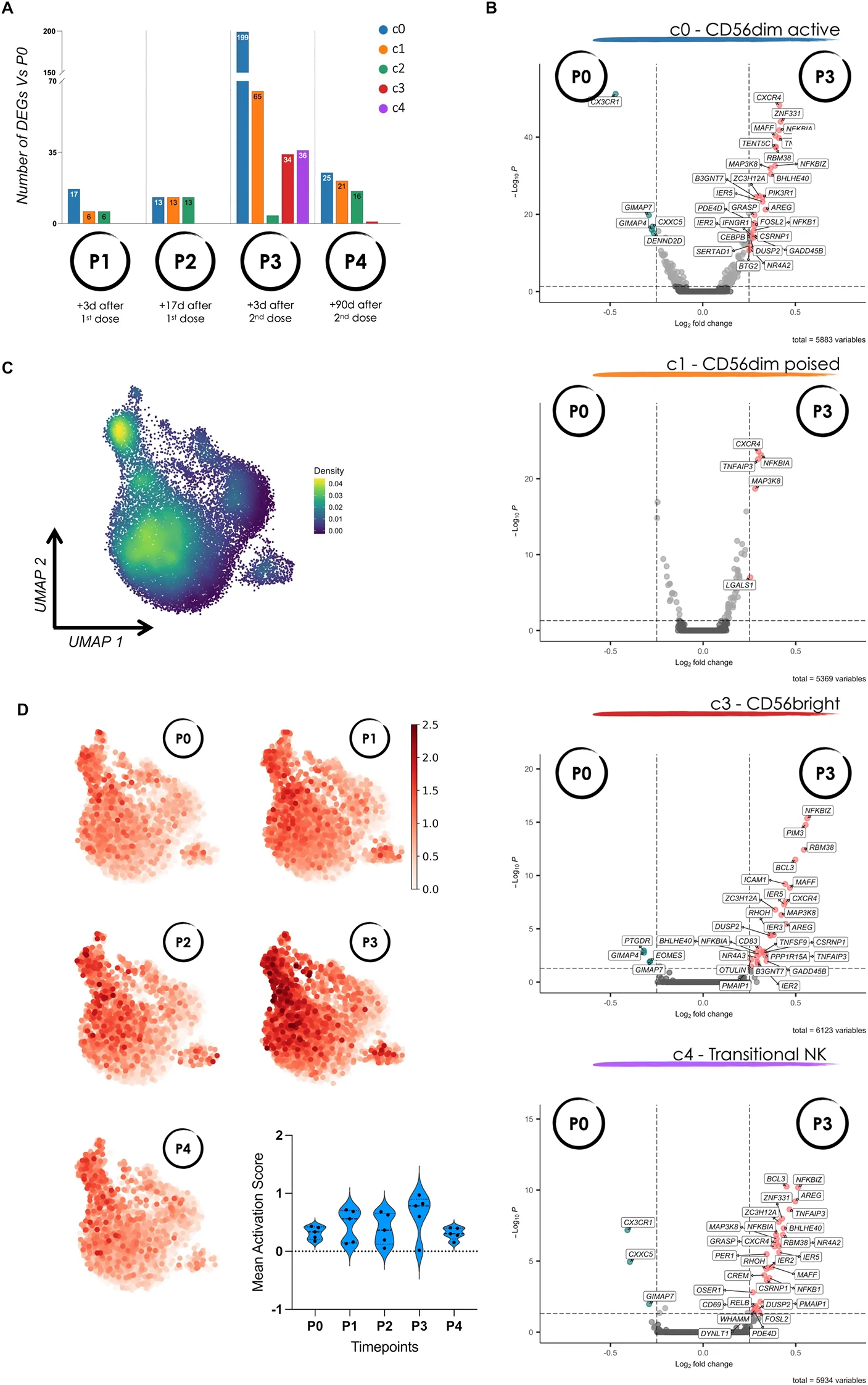
NK cells are more active after the booster dose of vaccine. **(A)** Bar plot showing the total number of identified DEGs with adjusted P values (adj.p) ≤ 0.05 across different time point (P1-P4) after vaccination, compared to pre-vaccine time point (P0). **(B)** Volcano plots showing the fold change and Log_10_P-value of DEGs of c0-c4 across time point P3 compared to P0. Red dots indicate genes up-regulated (right), blue dots the genes down-regulated (left) in P3 vs P0 with absolute value of average |Log2- FC| ≥ 0.25 and adj.p ≤ 0.05 Light gray dots indicate genes with adj.p ≤ 0.05 and a |Log2- FC| ≤ 0.25, while dark gray indicates not significant DEGs with adj.p≥0.05. **(C)** Kernel Density plot of the activation score embedded on a UMAP across all the time points. **(D)** Feature plots of activation score embedded on UMAPs split by different time points and violin plots showing the mean activation score across for each donor over time. Kruskal-Wallis test with multiple comparison; n.s.

To further characterize the immunological response elicited in NK cells by the second administration of the vaccine, we examined DEGs at P3 relative to the baseline (P0). We observed the upregulation of genes associated with NK cell activation, including *CD69* and several members of the NF-kB and AP-1 signaling cascade, across all cluster analyzed (**Figure 2B**). This comparison also highlighted differences between c0 and c1, with c0 displaying a transcriptional profile enriched in activation-associated genes, whereas c1 showed a comparatively less pronounced transcriptional response.

By combining the expression of several genes upregulated post booster administration, namely *NFKBIZ*, *IER2*, *IER5*, *IER3*, *DUSP2*, *NFKBIA*, *NFKB1*, *CD69* and *FOSL2*, we defined a vaccine-associated activation score that was subsequently applied for each cell and visualized on the UMAP plot. This score was highest in clusters c0, c3, and c4, particularly at P3, indicating enhanced expression of activation-associated transcripts at this time point (**Figures 2C, D**).

### 2.3 Integrated scRNA-seq analysis of NK cells in vaccinated individuals, SARS-CoV-2 acutely infected and convalescent patients resulted in the identification of 13 *bona fide* NK cell clusters

To gain a deeper insight into NK cell activation following vaccination and to delineate possible differences associated with natural infection, we compared the NK cell transcriptomic profiles of V group with those of subjects sampled during the acute COVID-19 (A) or after the spontaneous resolution of the infection (Convalescent, C) (36). Because longitudinal samples from the same COVID-19 patients were unavailable, we compared cross-sectional cohorts of acute and convalescent individuals with our longitudinal V group. Although this precludes within-individual tracking of NK-cell recovery, it enables the comparison of infection-associated states to vaccine-induced profiles. Among the 709’172 integrated high-quality PBMCs, NK cells were identified in c3-16-24-28-38 (**Supplementary Figures 2A, B**). In detail, c28 was annotated as CD56bright, whereas c3-16-24–38 were classified as CD56dim, by using the same approach described above (**Figure 2**).

Healthy donors from A and C datasets, and unvaccinated samples from the V cohort (P0) were grouped together as Naïve (N) group (**Figure 3A**).

**Figure 3:**
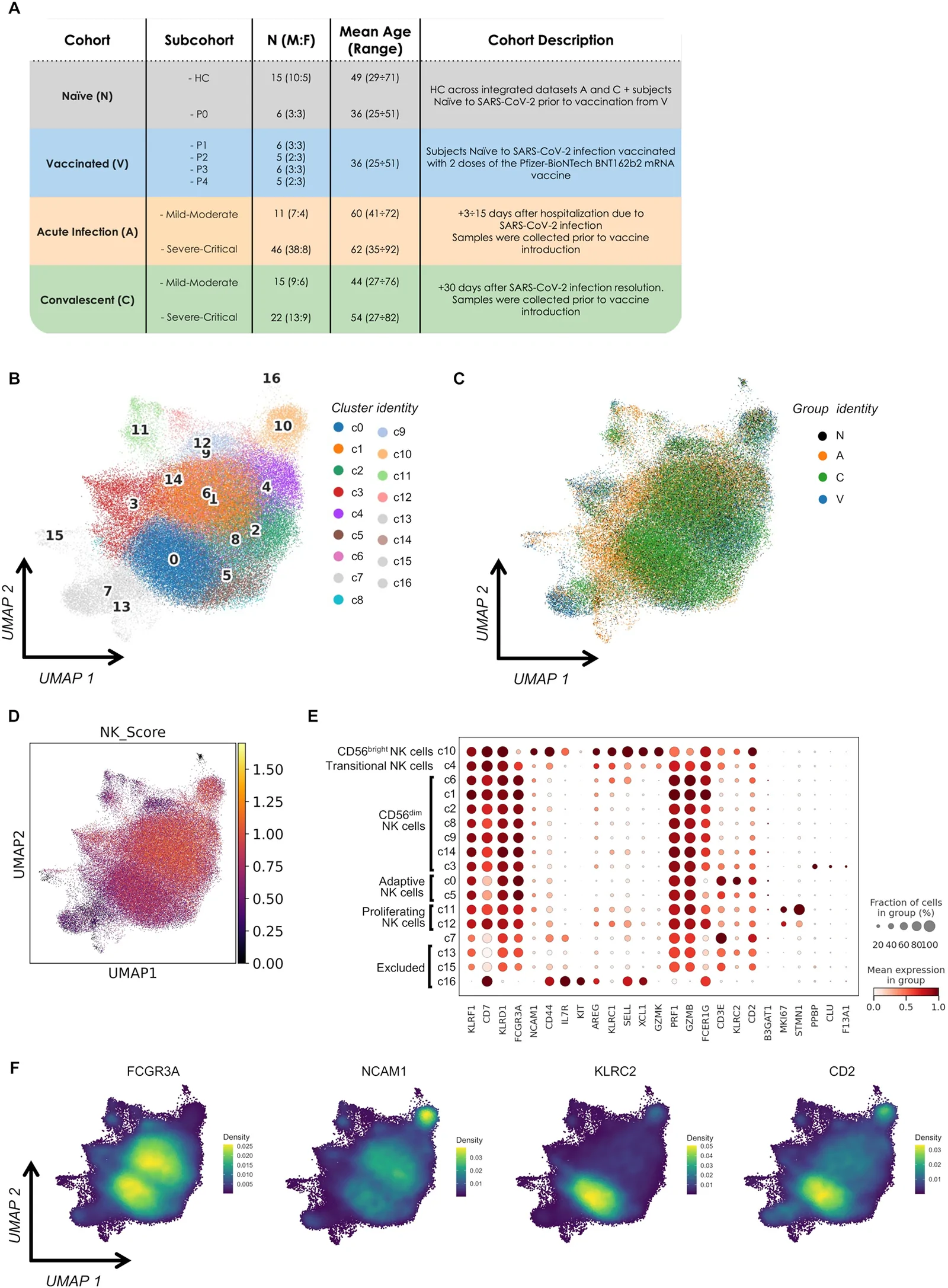
NK cell annotation of integrated N, V, A, C groups. **(A)** Descriptive table of the integrated cohorts. **(B, C)** UMAP visualization of the 88’580 integrated NK cells identified across the three datasets colored based on the **(B)** cluster or **(C)** dataset (Naïve (N), black; Acutely infected (A), orange; Convalescent (C), green; Vaccinated (V), blue) identity. **(D)** UMAP colored according to the NK cell score used to confirm the NK cell identity. **(E)** Dot plot showing the expression of genes used to annotate NK cell subsets within the different clusters of the integrated NK cells. Dot dimension represents the % of expression, while the color intensity indicates the mean gene expression. **(F)** Kernel Density plots showing the expression of the canonical markers used for NK cell annotation within the UMAP.

A total of 88’580 NK cells was subsequently reclustered, resulting in 17 clusters (**Figure 3B**), composed by NK cells shared among the four groups (**Figure 3C**). Prior to the annotation, we applied a recently described NK cell scoring system based on a 13-genes signature expressed by circulating NK cells (34). This score resulted particularly low in clusters c7, c13, c15 and c16, that were thus excluded from further analysis (**Figure 3D**). Residual contamination of other cell types was also assessed by plotting lineage markers. The clusters negative for the NK cell score showed, in addition to low levels of *CD7*, transcripts associated with unconventional T cells or circulating helper Innate lymphoid cells (not shown).

We next annotated the integrated NK cells manually. CD56bright NK cells were identified in c10 characterized by high expression of *NCAM1*, *CD44, IL7R, KIT, KLRC1, SELL, XCL1*, and *GZMK.* transitional NK cells were mapped to c4, displaying an intermediate profile between CD56bright and CD56dim. CD56dim NK cells were distributed across clusters c1-3, c6, 8, 9, and c14, and were defined by the expression of *FCGR3A*, *PRF1* and *GZMB.* Adaptive-like NK cells were localized in c0 and c5, characterized by the expression of *CD3E, KLRC2*, and *CD2.* Proliferating *MKI67, STMN1* NK cells were enriched in c11-12 (**Figure 3E**). Cluster annotations were further validated by Kernel Density plots based on major canonical markers expression to annotate NK cell subpopulations (**Figure 3F**).

### 2.4 SARS-CoV-2 infection alters NK cell composition

We next investigated the differences in NK cell composition across the various groups by applying a recently described set of signatures for NK1, NK2 and NK3 that helps in identifying major circulating NK cells populations (34). Briefly, the NK1 signature is characterized by high expression of markers of NK cell maturity as *KLRB1*, *FCER1G*, *CD247*, *CX3CR1* and cytotoxic mediators (*PRF1*, *GZMB*) and mapped the clusters that we annotated as CD56dim NK cells in our cohort. NK2 signature, characterized by the expression of immunoregulatory markers (*GZMK*, *XCL1*, *XCL2*, *AREG*), transcripts related to tissue homing (*SELL*, *CD44*), cytokine receptors (*IL7R*, *IL2RB*) and high expression of *KLRC1*, was specifically enriched in clusters c4 and c10 of CD56bright and transitional NK cells. Last, NK3 signature, characterized by a variety of CD3 transcripts (*CD3E*, *CD3G*, *CD3D*), *CD2*, *KLRC2* and by secreted factors like *GZMH* and *IL32*, strongly overlaps with clusters c0 and c5 of adaptive-like NK cells (**Figure 4A**).

**Figure 4:**
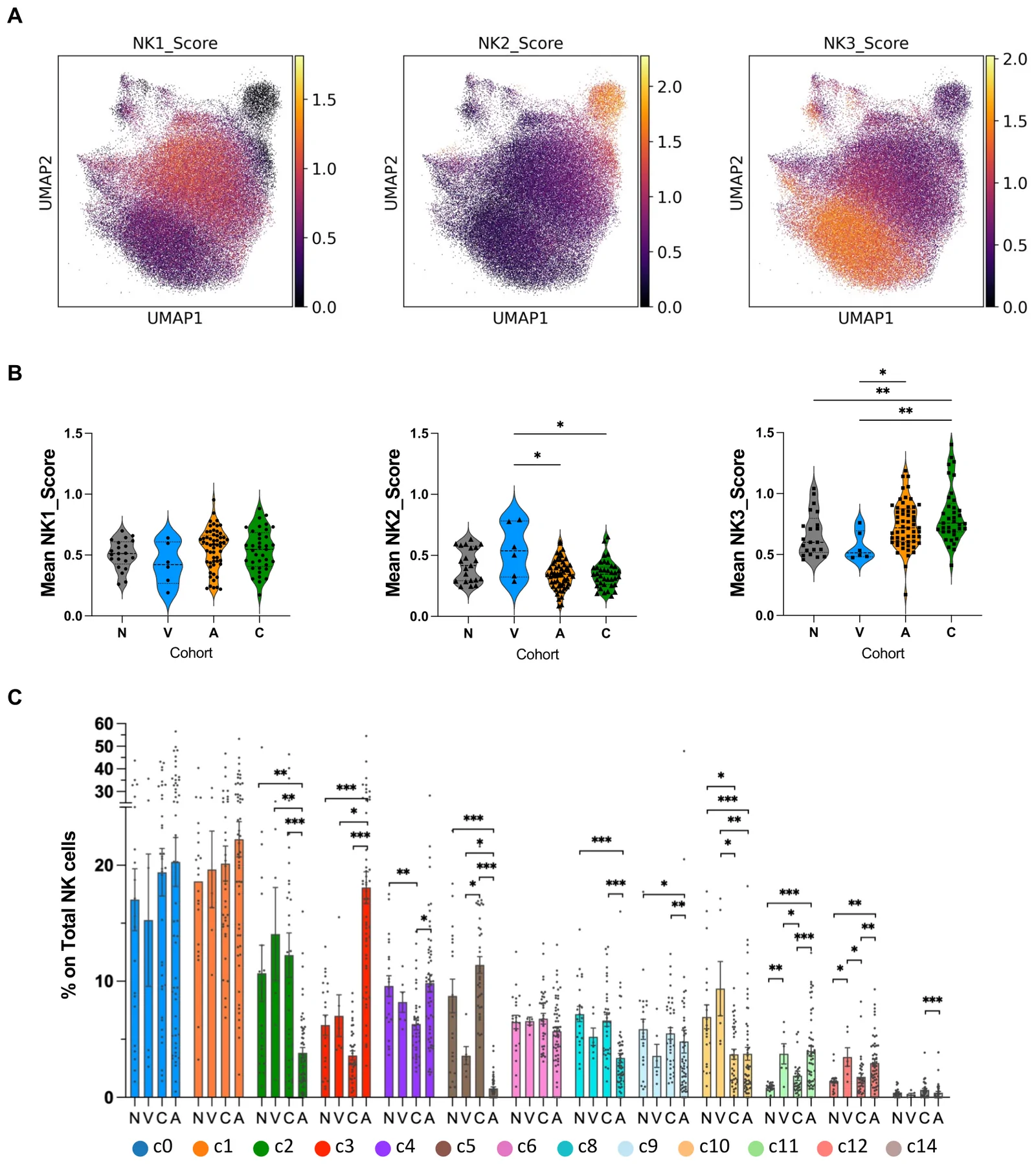
NK cell composition in N, V, A, C, groups. **(A)** UMAP colored according to NK1, NK2 and NK3 scores. **(B)** Violin plots showing the mean value of NK1, NK2 and NK3 scores across the different cohorts (N, Naïve; V, Vaccinated; A, Acutely Infected; C, Convalescent). Kruskal-Wallis test with multiple comparison; *p<0.05, **p< 0,005, ***p<0.001. **(C)** Bar plot showing the frequencies (%) of NK cell cluster distribution across N, V-P3, C and A groups. Data are presented as mean +/- SEM. Kruskal-Wallis test with multiple comparison; *p<0.05, **p< 0,005, ***p<0.001.

By assessing the differences in each NK cell score across our cohort, we demonstrated that the average level of the NK1 score was similar in all the 4 subject groups. On the contrary, NK2 was significantly reduced in both A and C groups compared to V at P3. Of note, both A and C groups were characterized by an increased NK3 score compared to V and N (**Figure 4B**). In both A and C groups, no differences were observed in NK2 and NK3 scores when comparing the disease severity within or between groups (not shown).

To further validate this observation, we investigated the frequencies of NK cell clusters within the 4 groups. V and N groups were not statistically different in the overall cluster composition, except for proliferating NK cells (c11, c12), that were expanded in V. Notably, this enrichment was observed only in the integrated analysis and was not detected in the longitudinal analysis of the vaccination cohort alone. Moreover, as c11 and c12 comprise proliferating cells derived from both NK1 and NK2 populations, they were not further considered in downstream analysis focused on bona fide NK-cell subsets. In contrast, the V group was characterized by the enrichment of c10 (CD56bright NK cells), which was conversely depleted in COVID-19 patients, both in acute and convalescent phases. The A group displayed a reshaping of the CD56dim NK cell compartment, with a significant expansion of c3 and a reduced frequency of c2. Last, the C group was specifically characterized by c5 (adaptive-like NK cells) with a frequency significantly higher than V and A (**Figure 4C**). Overall, these data support the emergence of distinct NK-cell compositional and transcriptional states following vaccination or natural infection and recovery.

### 2.5 Vaccination and acute SARS-CoV-2 infection elicited diverging transcriptional profiles in both CD56bright and CD56dim NK cells

First, we validated our vaccine-derived activation score by applying it on integrated NK cells, confirming its enrichment in c2 of CD56dim NK cells and c10 of CD56bright NK cells (**Figure 5**). Of note, both clusters are mainly expanded in the V group compared to A and C. Moreover, they appear to be particularly depleted in A, as previously shown (**Figure 4C**).

**Figure 5:**
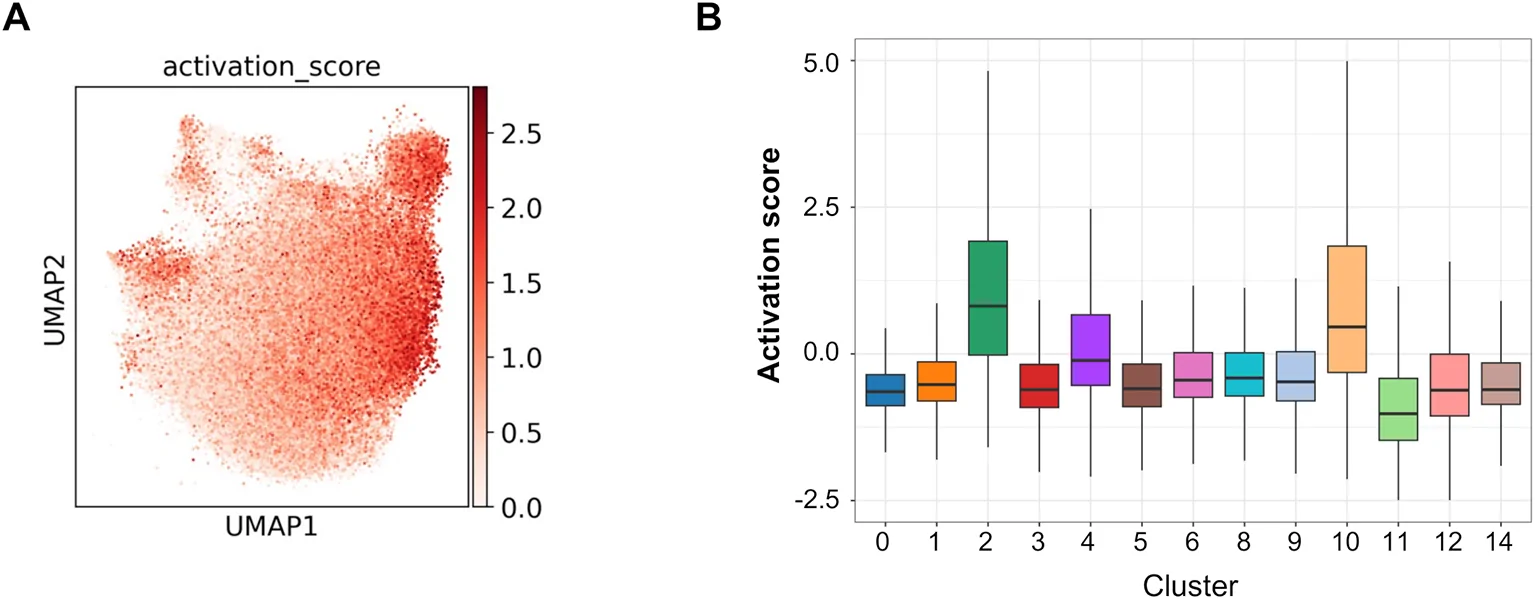
NK cell activation across the integrated dataset. **(A)** Kernel Density plot of the activation score embedded on the UMAP. **(B)** Boxplots showing the activation score for each cluster of all integrated NK cells.

To further investigate the cluster-specific transcriptional activation profiles throughout disease progression and in response to vaccination, we performed a differential gene expression analysis for c10 and c2 (**Figure 6**).

**Figure 6:**
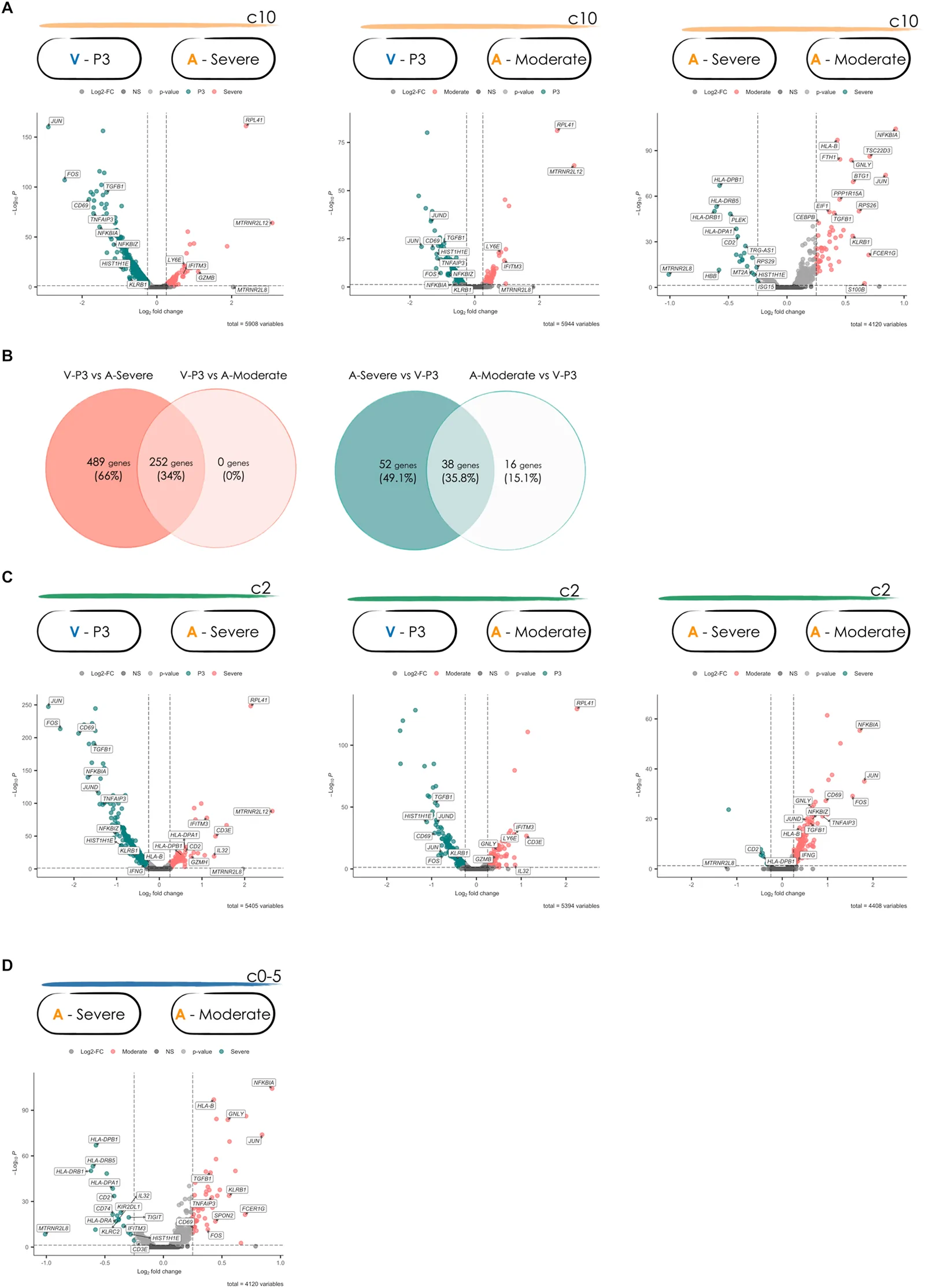
Comparison of DEGs in CD56bright, CD56dim and adaptive NK cells belonging to A and V groups. **(A)** Volcano plots highlighting DEGs of CD56bright NK cells in c10 in acutely infected patients with a severe disease (A-Severe) (left panel) and with a moderate disease (A-Moderate) (middle panel) compared to vaccinated subjects at time point P3 (V-P3), and in A-Moderate compared to A-Severe (right panel). **(B)** Venn diagrams showing the overlapping DEGs of CD56bright NK cells (c10) from the comparison between V-P3 and A group in severe and moderate conditions. Genes upregulated in V-P3 are reported in red (left panel), genes upregulated in A-Severe/Moderate in blue (right panel). **(C)** Volcano plots highlighting DEGs of CD56dim NK cells in c2 in A-Severe (left panel) and in A- Moderate (middle panel) compared to V-P3, and in A-Moderate compared to A-Severe (right panel). **(D)** Volcano plots highlight DEGs of adaptive-like CD56dim NK cells in c0 and c5 in A-Severe compared to A-Moderate. **(A, C, D)** Red dots indicate genes up-regulated (right), blue dots the genes down-regulated (left) with absolute value of average |Log2-FC| ≥ 0.25 and adjusted P values (adj.p) ≤ 0.05. Light gray dots indicate DEGs with average |Log2-FC| ≤ 0.25 or adj.p≥0.05. not-significant DEGs are depicted in dark gray.

By analyzing DEGs of c10 comparing V at P3 and A groups, we demonstrated that CD56bright NK cells from V bore a marked activation transcriptional signature compared to acutely infected individuals, regardless of the disease severity. Indeed, c10 of V group was characterized by the upregulation of the early activation marker *CD69*, and of various transcription factors, including genes of AP-1 (i.e., *JUN, JUNB, JUND, FOS, FOSB, FOSL2*), NF-kB (i.e., *NFKBIA, NFKBIZ, NFKB1*) families and members of MAPKs/DUSPs signaling cascade. On the contrary, many of the upregulated genes in moderate and severe acutely infected individuals were associated with canonical type-I IFN triggered responses (*LY6E, IFI6, IFI35, IFITM1, IFI44L*), Immediate Early Genes (IEGs), and genes related with viral infection (*MTRNR2L12*). Accordingly with recent studies (37, 38), our analyses also demonstrated that during acute infection NK cells display an increased expression of HLA genes, cytotoxic granules (*GZMA, GZMB, GZMH*, and PRF1), and of genes related to adaptive NK cell traits, namely *CD*2 and *CD3E* (**Figure 6A**).

Given the differences in the magnitude of DEGs in P3 versus the acute infection, hinting at a more diverging signature in severe patients with respect to their moderate counterparts, we also compared moderate and severe conditions within the A group. The data obtained suggest a more pronounced alteration of transcriptional functional programs in CD56bright NK cells from patients with a severe disease compared with those with a moderate disease (**Figure 6A**).

To further support our observation, we investigated the DEGs in V and A groups. Overall, analysis of the genes downregulated in A vs V revealed a core set of 252 genes shared between moderate and severe cases and additional 489 genes unique of the severe condition. Similarly, when examining the disease-specific upregulated genes, we identified a shared core of 38 genes between moderate and severe conditions, 52 genes exclusive to severe cases and only 16 exclusives of moderate cases (**Figure 6B**).

We next compared the DEGs of c2 (CD56dim NK cells) in V and A groups. The obtained data closely mirrored those of c10 both in terms of transcriptional changes and in the magnitude of the transcriptional alterations observed in acutely infected subjects, when comparing moderate and the severe forms of the disease (**Figures 6C, D**).

These findings encompass both the induction of IFN-stimulated mechanisms and granzymes, consistent with the previously reported association between type I IFN-related pathways and COVID-19 severity. Notably, like c10, CD56dim NK cells from both A-Moderate and A-Severe groups exhibited the upregulation of genes associated with an adaptive-like NK cell signature, namely *CD2*, *CD3E*, and *KLRC2.*

Given these observations, we next investigated the transcriptomic profile of adaptive-like NK cell clusters c0-5, comparing the acute and the severe conditions within the A group (**Figure 6E**). In line with previous results, adaptive-like NK cells from moderate patients displayed genes related to NF-kB and AP-1, as well as *CD69*, still retaining genes related to viral infection (*LY6E*). Of note, c0–5 NK cells of A-Severe patients showed a more pronounced expression of *CD2*, and *CD3E*, suggesting that transcriptional alterations observed in conventional NK cells from severe patients are also detectable within the adaptive-like NK-cell compartment. Furthermore, adaptive-like NK cells from A-Severe patients were characterized by the upregulation of the IL32 transcript, a key cytokine mainly secreted by this NK cell subpopulation, whose expression has been previously associated with COVID-19 severity (30, 39) (**Figure 6E**).

### 2.6 COVID-19 resolution partly restores NK cell transcriptional program

To better assess the long-lasting impact of COVID-19 natural infection on NK cells after the resolution of the disease, we last analyzed C subjects who had recovered from both moderate and severe infections, comparing them with their acutely infected counterparts. The data obtained demonstrated that, compared to A patients, C individuals display a profile more similar to that of CD56bright NK cells from vaccinated subjects, characterized by reduced expression of interferon-related genes and an increased expression of genes belonging to the AP-1 and NF-kB families (**Figure 7A**). This trend was observed in both C-moderate and C-severe, although severe infection appeared to induce a more pronounced and persistent impairment, as indicated by the higher number of DEGs. Similar results were obtained investigating the other NK cell subsets (not shown).

**Figure 7:**
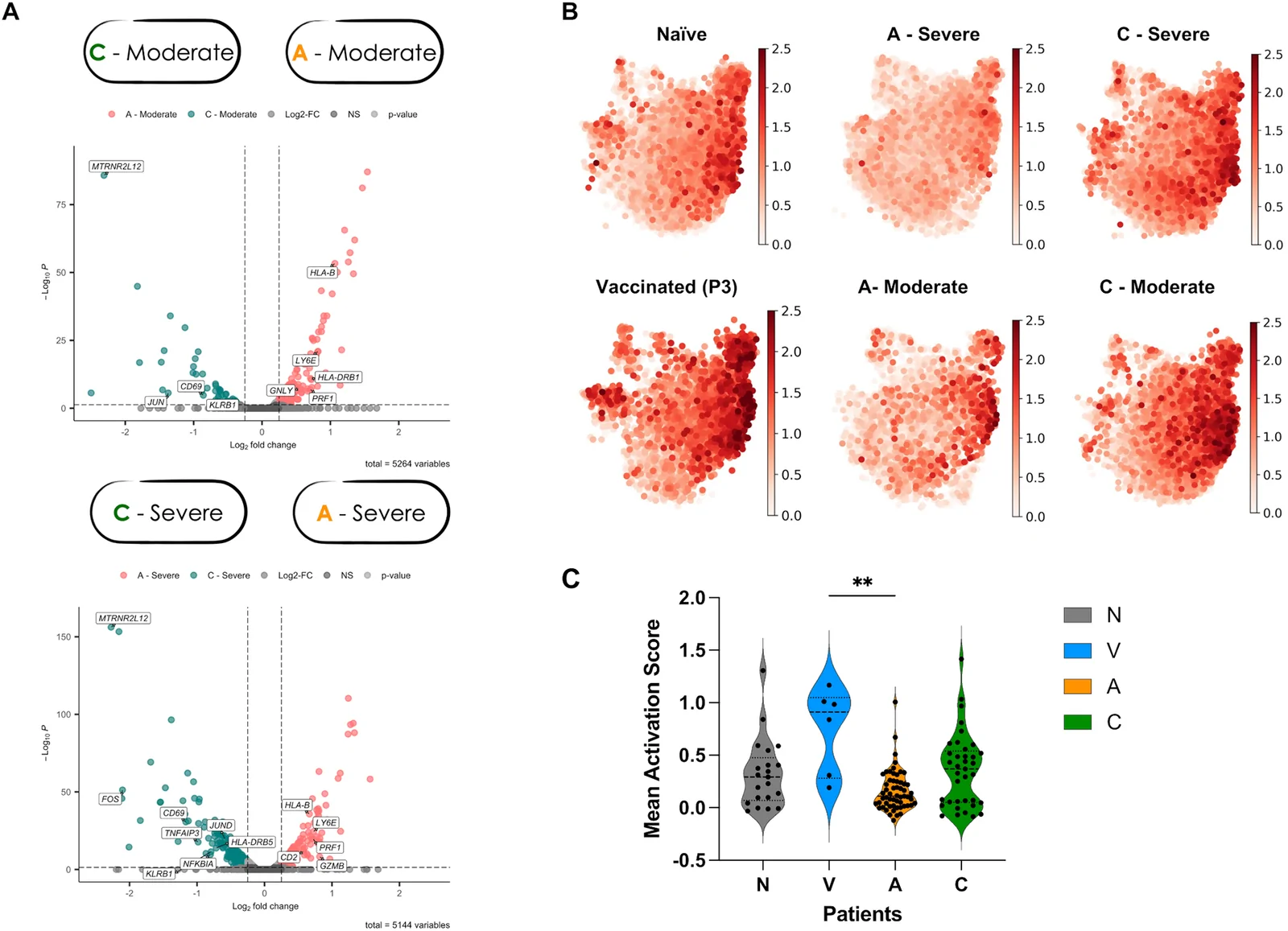
COVID-19 determines a long-lasting NK cell impairment especially in severe patients. **(A)** Volcano plots highlighting DEGs of CD56bright NK cells in c10 in convalescent patients who recovered from moderate disease (C-Moderate) compared to A-Moderate patients (upper panel), and in convalescent patients who recovered from severe disease (C-Severe) compared to A-Severe (lower panel). Red dots indicate genes up-regulated (right), blue dots the genes down-regulated (left) with absolute value of average |Log2-FC| ≥ 0.25 and adjusted P values (adj.p) ≤ 0.05. Light gray dots indicate DEGs with average |Log2-FC| ≤ 0.25 or adj.p≥0.05. not-significant DEGs are depicted in dark gray. **(B)** Activation score of each group embedded on the integrated NK cell UMAP. **(C)** Violin plot showing the activation score across the different groups of subjects; Kruskal-Wallis test multiple comparison, **p<0,005.

To further assess whether natural infection leads to lasting NK cell transcriptional modifications, we compared the DEGs of CD56bright NK cells in convalescent patients and vaccinated individuals at P4. Intriguingly, no differences in terms of NK cell activation were observed (not shown).

Collectively, these findings suggest a partial recovery of NK-cell transcriptional programs following COVID-19 resolution. However, transcriptional differences remained detectable in individuals who experienced severe disease. To further investigate this observation, we applied the activation score, previously defined in the V group, to patient cohort and stratified by disease severity. The results obtained support the hypothesis that acute infection, particularly in its severe form, is associated with the most pronounced changes in genes related to NK cell activation and effector programs, whereas the highest transcriptional activation score was observed in the V subjects 3 days after the booster administration (**Figures 7B, C**).

## 3 Discussion

NK cells are key innate effectors of anti-viral immunity. In the context of SARS-CoV-2 natural infection, several lines of evidence demonstrated that this coronavirus evades the NK cell-mediated immune response using a two-fold strategy: first, by impairing NK cell function via exhaustion, loss of activating receptors, and increased expression of the inhibitory receptors, such as NKG2A; and second, by reducing target cell susceptibility through the downregulation of ligands for NK cell activating receptors. This coordinated immune evasion reflects an evolutionary pressure and could contribute to the high pathogenicity of SARS-CoV-2 (13).

The huge heterogeneity in symptoms and clinical manifestation as well as the potentially fatal clinical outcome of SARS-CoV-2 infection, prompted the rapid development and application of mRNA vaccines, including BNT162b2, which elicit robust adaptive responses against the viral spike protein. However, the durability of this protection decreased overtime, requiring booster administrations. Although much attention has been devoted to B and T cell memory following vaccination, emerging evidence suggests a contribution of the early activation of NK cells in the generation of immunity induced by anti-SARS-CoV-2 mRNA vaccine (40). Furthermore, while the contribution of NK cells to natural SARS-CoV-2 infection has been widely investigated, their contribution and functional programming in response to mRNA vaccination remain poorly defined.

Herein, we address this gap by performing a longitudinal scRNA-seq analysis to characterize NK cell behavior in SARS-CoV-2 naïve individuals, who received two doses of the BNT162b2 vaccine. We further compared their transcriptional profiles with those of NK cells from acutely infected or convalescent COVID-19 patients, spanning different disease severities. This comprehensive approach enabled the identification of a vaccine-induced NK cell transcriptional programs and provided a framework to compare innate immune adaptations induced by vaccination versus natural infection.

Consistent with previous studies (30, 41), neither the first administration of the mRNA vaccine, nor the booster significantly altered the frequency of circulating NK cells or the distribution of NK cell subsets, which appeared stable over time. Indeed, most NK cells transcriptionally resembled the CD56dim subsets, followed by CD56bright and transitional NK cell subpopulations, as typical of the periphery. Despite this compositional stability, longitudinal transcriptional profiling revealed progressive changes in NK-cell transcriptional programs following sequential vaccine administrations, characterized by increased expression of genes correlated with NK cell activation, including transcription factors from the AP-1 and NF-kB families, involved in the upregulation of early activation genes, like *CD69* (41). Notably, whereas the first vaccine dose was associated with transcriptional changes primarily detectable in CD56dim NK-cell clusters, the booster dose was associated with a broader and more pronounced transcriptional response encompassing both CD56bright and transitional NK cells.

Together these data demonstrate that repeated antigen exposure is associated with progressively broader transcriptional changes in NK cells following mRNA vaccination. Notably, the booster dose was associated with a more pronounced activation-associated transcriptional signature than the primary immunization, involving a wider range of NK-cell subsets.

In line with recent evidence, these transcriptional changes raise the hypothesis that repeated vaccine doses may prime NK cells for enhanced responses, analogous to trained immunity (42), but functional validation is required.

NK cell function has been reported to be impaired during COVID-19, and has been associated with disease severity, although whether these NK-cell states casually contribute to pathogenesis or reflect disease-driven remodelling remains to be determined (13, 30). To delineate the transcriptional differences in the NK cell compartment induced by natural SARS-CoV-2 infection versus vaccination, we integrated our scRNA-seq dataset with data from acutely infected patients, experiencing either severe or mild-moderate COVID-19, and from convalescent individuals, who spontaneously resolved the infection.

This integrative analysis suggests that SARS-CoV-2 infection is associated with a remodeling of circulating NK cell composition. While the NK1/CD56dim overall frequencies were largely preserved across all groups, regardless of the disease severity and infection status, both acutely infected and convalescent individual exhibited a significant reduction in the frequency of NK2/CD56bright NK cells, coupled with a marked expansion of NK3/adaptive-like NK cells. These findings are consistent with previous reports associating adaptive NK cell expansion with severe COVID-19 outcomes (30, 35). Because acute and convalescent cohorts were cross-sectional, we cannot fully determine whether the observed NK-cell alterations in convalescent individual reflect infection-induced persistence or pre-existing differences, further longitudinal studies are required to assess the potential of SARS-CoV-2 to imprint long-lasting alterations in innate lymphocyte composition, that may persist beyond clinical resolution. In contrast, vaccination preserves NK cell homeostasis in terms of frequencies and subset composition, whereas the booster predominantly promoted the activation rather than compositional remodeling of NK cells.

To further dissect whether identical NK cell clusters might exhibit distinct activation profiles in the context of natural infection, we defined a transcriptional activation score integrating the expression of key early activation and immediate-early genes identified in the booster-versus-baseline comparison, including *CD69, FOSL2, NFKB1, NFKBIA, NFKBIZ, DUSP2, IER2, IER3 and IER5.* This approach enabled the identification of NK-cell clusters enriched for activation-associated transcriptional signatures across the integrated cohorts. The highest scores were observed in two populations, a cluster of activated CD56dim NK cells and a CD56bright NK-cell cluster, markedly reduced during acute infection. Comparative analysis of gene expression across these two clusters identified a transcriptional signature enriched for viral sensing and type I interferon-associated pathways that was predominantly represented in acutely infected patients and was more evident in severe disease (37). Consistent with previous reports (37, 43), NK cells from COVID-19 patients displayed increased expression of interferon-stimulated genes together with reduced expression of activation-associated transcripts. Although this alteration was more pronounced during the acute infection, it persisted in convalescent individuals, especially in those who experienced and recovered from severe disease, suggesting incomplete normalization of NK-cell transcriptional programs following recovery from severe COVID-19.

Concomitant with the reduced expression of activation-associated transcripts, we observed the upregulation of genes associated with the NK3/Adaptive NK cell signature both in the CD56bright and in the CD56dim NK cell clusters. Although the molecular cues governing differentiation towards NK3/Adaptive NK cells remain unclear, the increased expression of transcripts such as *CD3E*, *KLRC2* and *CD2* in COVID-19 patients may reflect a broader transcriptional reprogramming of NK cells rather than the acquisition of a bona fide adaptive-like differentiation program, which has been most clearly characterized in the context of HCMV infection (30, 38, 40, 44). This interpretation is further supported by the direct comparison of adaptive-like NK cell clusters across groups, which revealed sustained upregulation of these transcripts together with a marked increase of the IL-32 transcript, a cytokine implicated in the exacerbation of COVID-19 immunopathology, particularly in patients with severe forms of the disease (39, 44, 45). Collectively, these findings demonstrate an enrichment of adaptive-like NK cell transcriptional signatures both during the acute and convalescent phases of SARS-CoV-2 infection. The data support the presence of a shared NK3-related transcriptional program across COVID-19 patients, while the biological nature of this signature should be interpreted with caution, as it does not necessarily imply adaptive-like NK-cell expansion or functional impairment.

From a clinical perspective, the depletion of CD56bright NK cells and the reshaping of CD56dim NK cells observed in COVID-19 patients correlate with transcriptional signatures of interferon signaling and reduced activation markers, particularly in severe disease. Furthermore, the persistent dominance of adaptive-like NK cells observed in convalescent individuals raises the possibility that SARS-CoV-2 infection may be associated with sustained alterations in NK-cell composition beyond the acute phase of disease. In contrast, vaccination preserves NK cell homeostasis while promoting a controlled and transient activation program, highlighting a key functional advantage of vaccine-induced immunity.

A limitation of this study is that our conclusions are primarily based on transcriptomic profiling, which may not fully capture NK-cell functional states or effector responses at the protein level. Functional assays, including cytotoxicity and cytokine secretion analyses, and proteomic approaches will be therefore required to determine whether the identified transcriptional signatures correspond to altered NK-cell activation or dysfunction. In addition, the relatively small vaccination cohort (n = 6) limits the generalizability of our findings, and the cross-sectional design comparing acute and convalescent cohorts precludes within-individual assessment of NK-cell recovery dynamics over time. Furthermore, the activation score used in this study was derived from vaccination-induced responses and may not fully reflect activation states in infection settings, where interferon-driven programs can predominate. Finally, the lack of correlation with clinical parameters, such as viral load or recovery kinetics, limits the clinical interpretation of the identified NK-cell states.

Future studies integrating longitudinal sampling, larger cohorts, functional and proteomic analyses, and clinical outcome measures will be important to validate and extend these findings. Nevertheless, our data identify distinct NK-cell transcriptional signatures associated with vaccination and SARS-CoV-2 infection and provide a basis for future investigations exploring their biological relevance and potential clinical utility in developing NK cell-targeted strategies to enhance vaccine efficacy and highlight the potential of NK cell signatures as biomarkers to stratify immune protection and predict infection outcomes.

## 4 Materials and methods

### 4.1 Sex as a biological variable

Our study examined male and female individuals (**Figure 3A**), and sex was not considered as a key biological variable.

### 4.2 Subject enrollment and sample collection

This study was designed to evaluate the NK cell immune response in healthy individuals receiving mRNA COVID-19 vaccine (V group), unvaccinated individuals with acute natural infection (A group) and unvaccinated individuals in their convalescent phase (C group).

#### 4.2.1 V group

Healthy volunteers naïve for COVID-19 were enrolled during the Italian COVID-19 vaccination program at Humanitas Research Hospital (HRH) from November to April 2021.

This group included six volunteers naïve for SARS-CoV-2 infection who received two doses of Pfizer-BioNTech BNT162b2 vaccine in January 2021. Peripheral blood samples (n=28) were collected longitudinally 1 day before (P0, n=6), 3 (P1, n=6) and 17 (P2, n=5) days after prime vaccination, and 3 (P3, n=6) and 90 (P4, n=5) days following vaccine-boost (**Figure 1A**). Although all six volunteers were enrolled throughout the study period, one sample at P2 and one sample at P4 did not meet the quality criteria required for single-cell RNA sequencing because of reduced cell viability and were therefore excluded from downstream analysis. All individuals included in the study were asymptomatic (such as fever or cough) and did not receive any additional vaccines, throughout the vaccination period.

After blood collection, PBMCs were isolated through Lympholyte^®^-H Cell Separation density gradient solution (Cedarlane Laboratories, Burlington, North Carolina, USA) according to the manufacturer’s instruction. PBMCs were then frozen in Foetal Bovine Serum (FBS) supplemented with 10% of the cryoprotective Dimethyl Sulfoxide (DMSO) until use.

#### 4.2.2 A group

This group included 57 acutely infected patients PCR-positive for SARS-COV-2 enrolled between September 2020 and March 2022 at the University Hospital “L. Sacco”, the primary medical facility for managing COVID-19 cases during the pandemic in the Milan area of Italy. Peripheral blood samples were collected within 3–15 days from hospitalization. Patients were classified according to disease severity into two groups (**Figure 3A**): mild-moderate (n=11), including patients who did not require supplemental oxygen (mild) and patients with a modest oxygen requirement via nasal cannula (moderate), and severe-critical (n=46), including patients affected by respiratory deficiency requiring high-flow oxygen support and patients in respiratory failure who required continuous positive airway pressure (C-PAP) or mechanical ventilation. Patients included in this study were hospitalized before February 2021, prior to the introduction of SARS-CoV-2 vaccination and to minimize viral variability. Patients with prior corticosteroid treatment before hospitalization and those with underlying oncologic or immune disorders were excluded. PBMCs were isolated from by density gradient centrifugation by using Histopaque-1077 (Merck, Italy), according to standard procedures. Freshly isolated cells were immediately used.

#### 4.2.3 C group

Data and detailed clinical information were downloaded from gene expression omnibus (GEO) database under the accession number GSE158055 (36). This group comprises 37 convalescence patients, 22 recovered from severe disease and 15 from moderate disease (**Figure 3A**). PBMCs were collected approximatively 76 days (range 30–127 days) after SARS-CoV-2 infection. Patients were selected before the introduction of SARS-CoV-2 vaccines and according to similar inclusion and exclusion criteria of A group.

#### 4.2.4 Healthy controls

Fifteen HCs, 10 sequenced together with the A group and 5 belonging to the public database (GSE158055) naïve for SARS-CoV-2 infection and enrolled prior the availability of anti-COVID-19 vaccines were included as control. These HCs, together with the time point P0 of the 6 V individuals, were included in the Naïve group upon integration (**Figure 3A**).

### 4.3 scRNA library preparation

#### 4.3.1 V group

Libraries for scRNA-seq were prepared using the Chromium Single Cell Platform with a Single-Cell 5′ Library and Gel Bead Kit (10X Genomics, PE-1000006). Thawed PBMC samples were evaluated for viability before scRNA-seq analysis. Only samples with a viability higher than 95% were sequenced. Cells were resuspended in a volume equivalent to 10,000 target cells for each sample and were individually loaded onto a Chromium single-cell controller (10X Genomics) to generate single-cell gel beads-in-emulsion (GEMs). Captured cells were then lysed and the released RNA was barcoded through reverse transcription in individual GEMs. Complementary DNAs (cDNA) were generated and amplified; the quality was assessed using an Agilent 4200 Tapestation.

The scRNA-seq libraries were sequenced using an Illumina NextSeq 550 sequencer with a paired-end 150-bp (PE150) reading strategy (performed by CapitalBio Technology).

#### 4.3.2 A group

Single-cell PBMCs were labelled with the cocktail of oligo-bound antibodies TotalSeq-C Human Universal Cocktail, V1.0 (Biolegend) and encapsulated into Gel Emulsion Droplets using the Chromium Controller system (10x Genomics) targeting 5’000 cells for each individual.

Afterward, 5’ Gene Expression and feature barcode (for surface proteins) libraries were generated following the 10x Genomics Protocols. Libraries were pooled at a ratio dependent on the 10x Genomics indication and loaded on the Novaseq6000 sequencer to achieve a minimum of 50’000 reads/cell for the 5’ Gene Expression libraries.

### 4.4 Processing scRNA-seq data

For V and A datasets, the scRNA-seq reads were aligned to the GRCh38 (version refdata-gex-GRCh38-2020-A, 10X Genomics) human genome reference, and UMIs were quantified using Cell Ranger 5.0.0 (10X Genomics). Subsequent analyses were performed using the Python package Scanpy v1.8.1 under Python v3.8, if not stated otherwise. For the C group, the analyses were conducted starting from the count matrices provided in the GEO repository.

For all the datasets, cells were filtered out based on thresholds evaluated on the distribution of the counts per transcript, the total transcript count, and the percentage of counts of mitochondrial transcripts. For the A group, cells were also filtered based on the counts from antibody sequencing and their percentage of control antibodies to the total antibody counts when available. Specifically, only cells with at least at least 1’200 total counts, 600 expressed genes and 40 different cell surface proteins from CITE-Seq were kept for subsequent analysis. Cells were excluded from the analysis if having more than 8% of the overall reads of mitochondrial origin. Data were then log-normalized with a scale factor of 10’000 and the expression of the protein quantification was normalized using the centered log ratio transformation as implemented by the *NormalizeData()* function in Seurat (46). Highly variable genes (HVGs) were identified using the Seurat dispersion-based methods using the ‘sample_id’ as batch key and as flavor “seurat_v3”.

### 4.5 Data integration

For the V group, principal component analysis (PCA) was performed using HVGs via ARPACK implementation of singular value decomposition. To limit the risk of biases, multiple sample-data integration was performed using the harmonypy package v0.0.677. More in detail, the 28 samples (labelled with a progressive sample_id name) were integrated using the sample_id as key covariate in the formula. Neighbors were identified using the top 50 components of Harmony-corrected PCA embeddings and clustering was performed using the Leiden algorithm (47) with an initial resolution of 0.1. Clusters were then embedded in two dimensions by using UMAP. Identified clusters were subjected to the next round of dimensionality reduction and unsupervised clustering, as described above. 10 clusters were identified at resolution 0.8, c9 was excluded from downstream analysis due to low expression of NK cell markers.

For the integration of all groups, a totalVI model (48) was trained from the highly variable genes count and the counts from the Ab-seq to obtain a variational autoencoder imposing a normal latent distribution and setting the ‘run_id’ as batch key. The model was used to obtain the joint latent representation from which a k-nearest neighbors’ graph was constructed using the Scanpy function *sc.pp. neighbors()*, imposing “n_neighbors = 10”. Leiden clustering using resolution 1 was performed on this graph. For visualization purposes, a UMAP was produced with the Scanpy function *tl.umap()*. Identified clusters were subjected to the next round of dimensionality reduction and unsupervised clustering, resulting in 17 clusters embedded on the UMAP.

NK cells were profiled and annotated based on known NK cell signatures and the expression of canonical cell-type markers, identified using the Wilcoxon rank-sum test and manual annotation. Genes with an FDR-adjusted P-value < 0.05, expressed in at least 10% of cells in the cluster, and with a minimum Log2-Fold Change of |0.25| were considered significant.

### 4.6 Differential gene expression analysis

Differential expression analysis between different time points and disease severity was performed to investigate gene expression differences using the *FindMarkers()* function provided by Seurat, with the negative binomial generalized linear model, and setting the latent variable as ‘Datasets’ for the integrated cohort. Genes with an FDR-adjusted p-value < 0.05, expressed in at least 10% of cells in the cluster, and with a minimum |Log2-Fold Change| of 0.25 were considered significant and visualized in a Volcano plot.

### 4.7 Activation score

The activation score was calculated based on activation signature (*NFKBIZ*, *IER2*, *IER5*, *IER3*, *DUSP2*, *NFKBIA*, *NFKB1*, *CD69*, *FOSL2*) obtained by selecting the activation-related genes among the DEGs in P3 versus the baseline and computed on NK cells from V dataset and on the integrated dataset using the *AddModuleScore()* function provided by Seurat.

### 4.8 Statistical analysis

GraphPad PRISM (version 10.0) and R statistical softwares were used. Different variables among samples were compared by Kruskall-Wallis test.

P values were considered significant when P ≤ 0.05 (*P < 0.05; **P < 0.01; ***P < 0.001; and ****P < 0.0001).

## 5 Resource availability

### 5.1 Lead contact

Requests for further information and resources should be directed to and will be fulfilled by the lead contacts, Clara Di Vito (clara.divito@unimi.it, clara.di_vito@humanitasresearch.it) and Domenico Mavilio (domenico.mavilio@unimi.it, domenico.mavilio@humanitas.it).

### 5.2 Study approval

The study protocol was in accordance with the ethical guidelines established in the Declaration of Helsinki, and participants signed written informed consent to participate in the study before taking part.

#### 5.2.1 V group

The collection of SARS-CoV-2 vaccinated subjects for research purposes has been ethically approved by the Institutional Review Board of HRH, Rozzano, Milan Italy (approval 738/20).

#### 5.2.2 A group

The study protocol was approved by the Institutional Review Board of University “L. Sacco” Hospital (approval 15725/2020 and following amendments).

## Data Availability

All data relevant to the study are included in the main text or in the Supplementary materials. The scRNA-seq data are available from the Gene Expression Omnibus repository (https://www.ncbi.nlm.nih.gov/geo/), accession code GSE260763 (V group), GSE158055 (C group) or from Zenodo (A group, doi: 10-5281/zenodo.23019755). R and Python scripts enabling the main steps of the analysis are available upon request to the corresponding authors.
