# Supplementary material for: Single-cell profiling of dysfunctional NK cells in COVID-19: from immunization to acute infection and beyond
Source: Front Immunol. 2026 Oct 20;17:1972608. doi: 10.3389/fimmu.2026.1972608 (PMC13727542; doi:10.3389/fimmu.2026.1972608)
Supplement: Supplementary file 1 [file DataSheet1.pdf]

Single-cell Profiling of Dysfunctional NK cells in COVID-19: from Immunization to Acute Infection and Beyond

Alessandro Frigo,[1,2] Sara Terzoli,[1,3] Paolo Marzano,[1] Nicolò Coianiz,[1] Michela Calvi,[1,2] Lara Orlandi,[1,2] Simone Balin,[1] Valentina Cazzetta,[1] Anna Carletti,[1] Silvia Della Bella,[1,2] Joanna Mikulak,[1] Maciej Tarkowski,[4] Matteo Bonfanti,[5] Paolo Ferrari,[5] Eugenia Ricciardelli,[5] Clelia Peano,[5,6] Agostino Riva,[4,7] Giuseppe Testa,[5,8] Antonio Voza,[3,9] Francesca Calcaterra,[1] Clara Di Vito[1,2]†*, Domenico Mavilio[1,2]†*

[1]Unit of Clinical and Experimental Immunology, IRCCS Humanitas Research Hospital, Rozzano, Milan, Italy.

[2]Department of Medical Biotechnology and Translational Medicine, University of Milan, Milan, Italy.

[3]Department of Biomedical Sciences, Humanitas University, Pieve Emanuele, Milan, Italy;

[4]Luigi Sacco Department of Biomedical and Clinical Sciences DIBIC, University of Milan, Milan, Italy.

[5]Human Technopole, Milan, Italy.

[6]Institute of Genetics and Biomedical Research, UoS of Milan, National Research Council, Milan, Italy.

[7]Department of Infectious Diseases, Luigi Sacco University Hospital, ASST Fatebenefratelli Sacco, Milan, Italy.

[8]Department of Oncology and Hemato-Oncology, University of Milan, Milan, Italy.

[9]Department of Biomedical Unit, IRCCS Humanitas Research Hospital, Milan, Italy.

† These authors contributed equally to this work

***Corresponding authors**:

Clara Di Vito

clara.divito@unimi.it; clara.di_vito@humanitasresearch.it

Domenico Mavilio

domenico.mavilio@unimi.it; domenico.mavilio@humanitas.it

1 Supplementary Figures

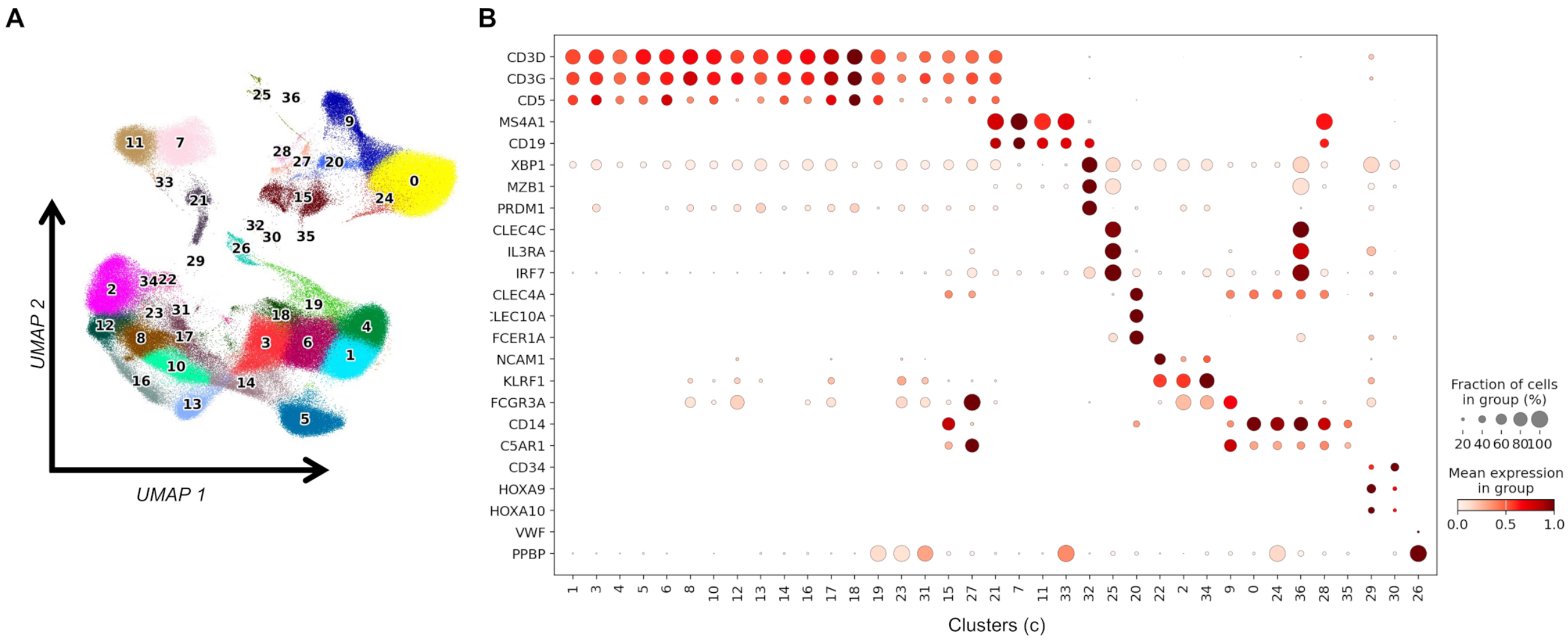

Supplementary Figure 1. NK cell identification within PBMCs of SARS-CoV-2-vaccinated subjects.

(A) UMAP visualization of the integrated PBMCs (242’765 cells) across all time points (P0-P4) and subjects (s01-s06) after quality control (QC) filtering. (B) Dot plot showing the expression of canonical markers used for the annotation of immune cell populations. NK cell clusters, c2, c22, and c34, were identified based on the expression of NK cell markers (*NCAM1*, *KLRF1*, and *FCGR3A*) and the absence of other lineage markers.

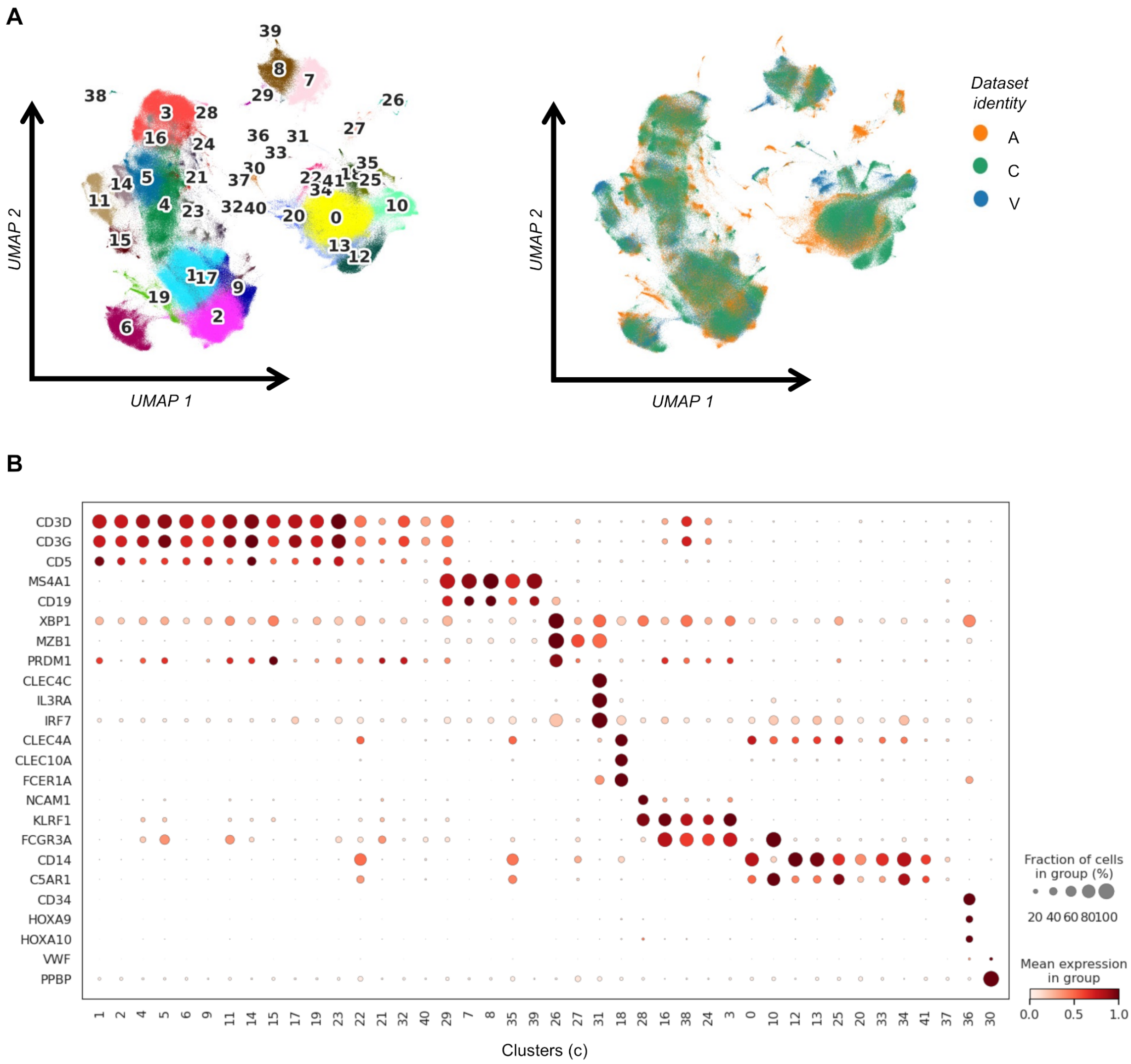

Supplementary Figure 2. NK cell identification in PBMCs after integration of acute infection, convalescent, and vaccination datasets.
(A) UMAP visualization of all integrated PBMCs (709’172) identified upon integration of all the three datasets (acutely infected, A; convalescent, C; and vaccinated, V) after QC filtering (left panel) and colored by its dataset identity (A, orange; C, green; V, blue; right panel).
(B) Dot plot showing the expression of canonical markers used for the annotation of all immune cell populations. NK cell clusters were identified based on the expression of known markers (*NCAM1*, *KLRF1*, and *FCGR3A*) in c3, c16, c24, c28, and c38.
